# Cross-cultural adaptation and psychometric evaluation of the Juvenile Arthritis Multidimensional Assessment Report (JAMAR) in 54 languages across 52 countries: review of the general methodology

**DOI:** 10.1007/s00296-018-3944-1

**Published:** 2018-04-07

**Authors:** Francesca Bovis, Alessandro Consolaro, Angela Pistorio, Marco Garrone, Silvia Scala, Elisa Patrone, Mariangela Rinaldi, Luca Villa, Alberto Martini, Angelo Ravelli, Nicolino Ruperto, For the Paediatric Rheumatology International Trials Organisation (PRINTO)

**Affiliations:** 1 Clinica Pediatrica e Reumatologia, Paediatric Rheumatology International Trials Organisation (PRINTO), Istituto Giannina Gaslini, Via Gaslini 5, 16147 Genoa, Italy; 2 Dipartimento di Pediatria, Università di Genova, Genoa, Italy; 3 Istituto Giannina Gaslini, Servizio di Epidemiologia, Biostatistica e Comitati, Genoa, Italy; 4 Istituto Giannina Gaslini, Direzione Scientifica, Genoa, Italy

**Keywords:** Juvenile Arthritis Multidimensional Assessment Report (JAMAR), Cross-cultural adaptation and psychometric evaluation methodology, Juvenile idiopathic arthritis (JIA), Healthy children, Review

## Abstract

The aim of this project was to cross-culturally adapt and validate the Juvenile Arthritis Multidimensional Assessment Report (JAMAR) questionnaire in 54 languages across 52 different countries that are members of the Paediatric Rheumatology International Trials Organisation (PRINTO). This effort was part of a wider project named Epidemiology and Outcome of Children with Arthritis (EPOCA) to obtain information on the frequency of juvenile idiopathic arthritis (JIA) categories in different geographic areas, the therapeutic approaches adopted, and the disease status of children with JIA currently followed worldwide. A total of 13,843 subjects were enrolled from the 49 countries that took part both in the cross-cultural adaptation phase and in the related validation and data collection: Algeria, Argentina, Belgium, Brazil, Bulgaria, Canada, Chile, Colombia, Croatia, Czech Republic, Denmark, Ecuador, Egypt, Estonia, Finland, France, Georgia, Germany, Greece, Hungary, India, Islamic Republic of Iran, Israel, Italy, Latvia, Libya, Lithuania, Mexico, Netherlands, Norway, Oman, Paraguay, Poland, Portugal, Romania, Russian Federation, Saudi Arabia, Serbia, Slovakia, Slovenia, South Africa, Spain, Sweden, Switzerland, Thailand, Turkey, Ukraine, United Kingdom and United States of America. 9021 patients had JIA (10.7% systemic arthritis, 41.9% oligoarthritis, 23.5% RF negative polyarthritis, 4.2% RF positive polyarthritis, 3.4% psoriatic arthritis, 10.6% enthesitis-related arthritis and 5.7% undifferentiated arthritis) while 4822 were healthy children. This introductory paper describes the overall methodology; results pertaining to each country are fully described in the accompanying manuscripts. In conclusion, the JAMAR translations were found to have satisfactory psychometric properties and it is thus a reliable and valid tool for the multidimensional assessment of children with JIA.

## Introduction

In recent years, there has been an increasing interest in parent/patient-reported outcomes (PROs) in juvenile idiopathic arthritis (JIA). Inclusion of these measures in patient assessment is deemed important as they reflect the parents’ and children’s perception of the disease course and effectiveness of therapeutic interventions. It has been suggested that PROs should be incorporated into routine paediatric rheumatology care. A number of measures for the assessment of PROs in children with JIA have been developed over the years, including questionnaires for the evaluation of functional ability and health-related quality of life (HRQoL), and visual analogue scales for parent/patient rating of well-being and pain [1]. However, most of these measures have remained essentially research tools and are not routinely administered in most paediatric rheumatology centres.

A multidimensional questionnaire was developed for the assessment of children with JIA in standard clinical care, which incorporates the traditional PROs (functional ability, HRQoL, overall well-being, pain) and other PROs such as morning stiffness, rating of disease course over time, proxy- or self-assessment of joint involvement, description of side effects of medications, and assessment of therapeutic compliance and satisfaction with the outcome of the illness. Administration of this questionnaire can provide physicians with a thorough and systematic overview of the patient status to be scanned at the start of the visit either on paper or online tools in the currently ongoing PRINTO academic projects. This facilitates focus on matters that require attention, leading to more efficient and effective clinical care. This tool is named Juvenile Arthritis Multidimensional Assessment Report (JAMAR) [2]. A parent proxy-report version, a child and adult self-report version of the JAMAR is available for use either on paper or on electronic devices. While the parent proxy-report and the child versions are fully cross-culturally adapted in several languages and validated as part of this supplement, the adult versions, which required modification in just few words, have been only cross-culturally adapted to accommodate participants as they matured into young adulthood. In the Istituto Giannina Gaslini clinic and in other paediatric wards where it is routinely used for the on-going PRINTO academic projects, the JAMAR has been found very user-friendly, easy to understand, and readily responded by parents and patients. It is quick, taking 5–10 min to complete and can be scanned by a health professional for a clinical overview in a few seconds, especially the electronic version, which summarises in few lines the most relevant findings either to health professionals and families/patients. Some components of the JAMAR have been already published in separate papers [3–5].

The widespread membership of the Paediatric Rheumatology International Trials Organisation (PRINTO at https://www.printo.it with more than 60 countries) [6] and the growing international collaboration along with the growing numbers of foreign patients requires the availability of the JAMAR cross-culturally adapted and validated according to international guidelines [7].

The aim of the present manuscript is to report the overall methodology for the cross-cultural adaptation and validation of the JAMAR into the languages of the countries belonging to PRINTO that agreed to be part of this international initiative.

## Methods

### Patients

Centres belonging to PRINTO were invited to participate. The validation of the JAMAR is part of a wider project named EPidemiology, treatment and Outcome of Childhood Arthritis (EPOCA) [8], which has the goal to obtain information on the frequency of JIA subtypes in different geographic areas, the therapeutic approaches followed by paediatric rheumatologists from diverse countries or continents, and the current disease and health status of children with JIA followed worldwide. Additional objectives are to investigate the availability of biologic medications in different countries to foster the regular use of standardized quantitative clinical measures in the clinical assessment of children with JIA in standard clinical care, and to promote the embracement of a uniform set of outcome measures across international paediatric rheumatology centres [8]. In brief, the National Coordinators of PRINTO (list at https://www.printo.it) were asked to coordinate the translation procedures (see below). To ensure that the data regarding the epidemiology, treatment and outcome of JIA are reliable, the collection of a representative sample of the patients followed at each participating centre was planned. Additionally, each centre was asked to enrol 100 unselected and possibly consecutive patients meeting the International League of Associations for Rheumatology (ILAR) criteria for JIA or, if the centre did not expect to see at least 100 patients within 6 months, to enrol all consecutive and unselected patients meeting the same criteria seen within the first 6 months after the study start.

The protocol was approved by the institutional ethics committees of the participating paediatric rheumatology centres as required by the national laws of each country. Parents/guardians/patients provided written informed consent to participate in the study.

Standard forms for data collection were designed using consensus methodologies at the PRINTO international Coordinating Centre in Genoa, Italy.

Patients with all JIA categories according to the ILAR criteria [9] were included in the study. All patients underwent clinical, rheumatologic, laboratory assessments and completed the JAMAR to evaluate the current status, including

- the physician’s evaluation of current disease activity on a 21-circle visual analogue scale (VAS);
- the parental assessment of overall well-being on a 21-circle VAS;
- the number of joints with active arthritis;
- the number of joints with swelling, pain, and limited range of motion;
- the C-reactive protein (CRP) and the erythrocyte sedimentation rate (ESR) (Westergren method).

Healthy controls were recruited from local schools (children from 6 to 18 years of age) and among the healthy brother(s) and sister(s) of the JIA children attending the clinics. A child was defined as healthy after examination by a physician and/or based on the parent’s declaration. Healthy controls completed just the JAMAR.

#### The questionnaires

A parent or legal guardian of each patient seen at the study units from October 2010 to August 2017 who was < 18 years was asked to complete the parent version of the JAMAR at each visit. On the same occasion, the child was asked to complete independently the patient version of the JAMAR. Methodology for the JAMAR development followed the FDA conceptual framework for developing a PRO instrument [10] and has been previously reported [2–5].

The JAMAR [2] includes the following 15 sections:

1. Assessment of physical function (PF) using 15 items in which the ability of the child to perform each task is scored as follows: 0 = without difficulty, 1 = with some difficulty, 2 = with much difficulty, 3 = unable to do. If it was not possible to answer the question or the child was unable to perform the task due to their young age or to reasons other than JIA the score was: not applicable. The total PF score ranges from 0 to 45 and has three components: PF-lower limbs (PF-LL); PF-hand and wrist (PF-HW) and PF-upper segment (PF-US) each scoring from 0 to 15 [3]. Higher scores indicate higher degree of disability [11–13]
2. Rating of the intensity of the patient’s pain on a 21-numbered circle visual analogue scale (VAS) (0 = no pain; 10 = very severe pain) [4];
3. assessment of the presence of joint pain or swelling in the following joints or joint groups: cervical spine, lumbosacral spine, shoulders, elbows, wrists, small hand joints, hips, knees, ankles, and small foot joints (present/absent for each joint);
4. assessment of morning stiffness (present/absent) and duration of morning stiffness;
5. assessment of extra-articular symptoms (fever and rash) (present/absent);
6. rating of the level of disease activity on a 21-circle VAS (0 = no activity; 10 = maximum activity) [4]. Although the ability of parents/patients to understand the meaning and to be able to report the extent of disease activity may be questionable, we decided to include this VAS to investigate whether it could be a better indicator of the level of disease activity than the well-being VAS. The latter scale has been found to reflect the effects of both disease process and damage, particularly in patients with long-lasting disease [4];
7. rating of disease status at the time of the visit (remission, continued activity or relapse);
8. rating of disease course from previous visit (much improved, slightly improved, stable, slightly worsened or much worsened);
9. checklist of the medications the patient is taking (list of choices);
10. checklist of side effects of medications;
11. report of difficulties with medication administration (list of items);
12. report of school/university/work problems caused by the disease (list of items);
13. assessment of HRQoL using a ten-item scale through the Physical Health (PhH), and Psychosocial Health (PsH) subscales (five items each) and a total score. The four-point Likert response, referring to the prior month, is ‘never’ (score = 0), ‘sometimes’ (score = 1), ‘most of the time’ (score = 2) and ‘all the time’ (score = 3). A ‘not applicable column was included in the parent version of the questionnaire to designate questions that could not be answered because of developmental immaturity. The total HRQoL score ranges from 0 to 30, with higher scores indicating worse HRQoL. A separate score for PhH and PsH (range 0–15) can be calculated [14–16]
14. rating of the patient’s overall well-being on a 21-numbered circle VAS;
15. a question about satisfaction with the outcome of the illness (Yes/No) [17].

The JAMAR is available in three versions, one for parent proxy-report (child’s age 2–18), one for child self-report, with the suggested age range of 7–18 years, and one for adult patients.

### Outline of the methods

**Fig. 1:**
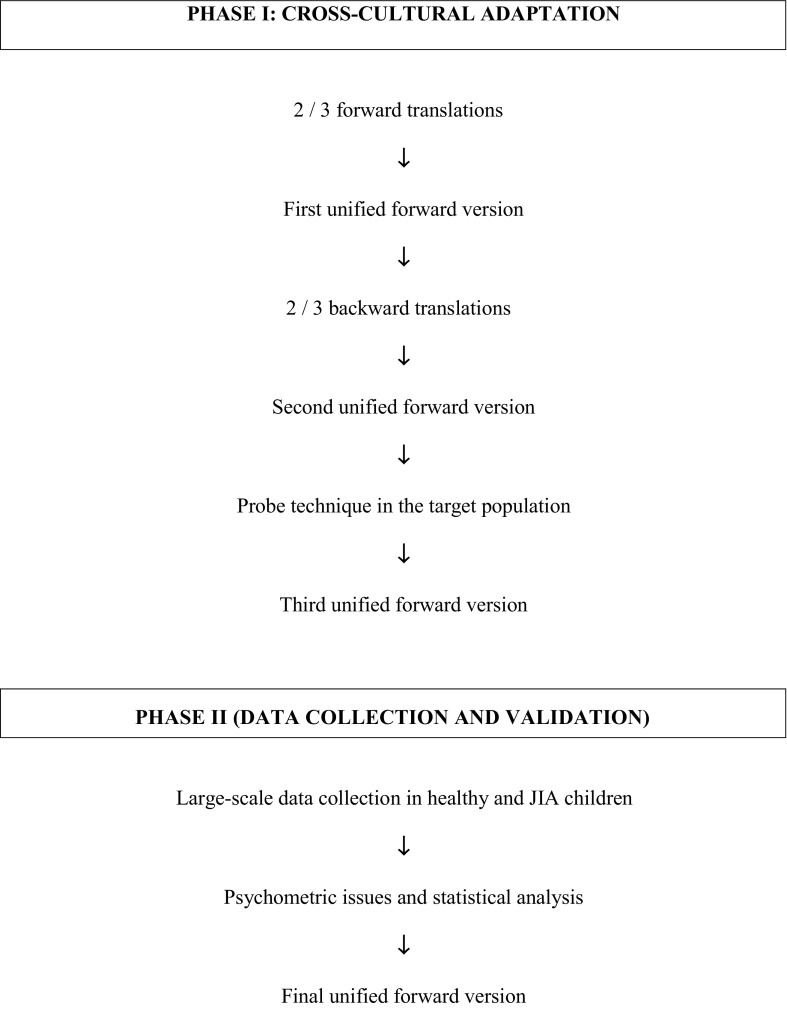
Diagram summarising the steps followed for the cross-cultural adaptation and validation procedures

The PRINTO project was divided into two phases (Fig. 1): phase I, the cross-cultural adaptation, which involved the translation procedures and preliminary probe in the target population; and phase II, the validation, which consisted of large scale data collection for psychometric and statistical evaluation.

#### Phase I: cross-cultural adaptation

The process of cross-cultural adaptation followed the guidelines provided by Guillemin et al. [18].

To facilitate comparisons among the different languages, the three versions of the questionnaire were, respectively, divided as follows: 123 lines of translations for the parent version of JAMAR and 120 lines of translations for child/adult version of JAMAR.

##### Forward translations into national languages

At least two (preferably three) literal translations from English have been done by 2–3 independent translators into their native language. The 2–3 translators were fluent in English and in the local language, were of different educational levels, background and sex, were instructed to use wording that could be understood by a 8–10-year old child (for the patient version of the JAMAR), and at least one of them was unaware of the purpose of the translations (e.g. should not be health professionals).

##### First unified forward translation

The 2–3 forward translators and 1 or 2 other persons not involved in the translation procedures (e.g. the local principal investigator and a nurse/physical therapist), met each other to reach a consensus (that was to reconcile differences in the forward translations) among the members of the group to obtain a first unified version from the 2–3 forward translations.

##### Backward translations into English

The first unified version of the questionnaires was back-translated by at least two (preferably three) independent backward translators with English as their first language and familiar with the idioms and colloquial form of the source national language. The 2–3 backward translators must not have taken part in the previous step. Backward translation is aimed at improving the quality of the final version of the questionnaires, by pointing out any misunderstandings of the first translation. Backward translators were unaware of the purpose of the concepts underlying the material and were of different educational level, background and sex.

##### Review of backward translations

The 2–3 backward translations were reviewed by PRINTO, whose main task was to check their correspondence with the original English version.

The aim of this phase was to make sure that the introductory material and the instructions for the translation of the questionnaires were still relevant based upon the final version of the translations, that the new translated JAMAR was fully comprehensible, and finally to verify its cross-cultural correspondence with the source text, by comparing their semantic, idiomatic, experiential and conceptual equivalencies.

##### Second unified forward version

A second meeting was then convened among all the forward and backward translators to take into account the comments received from the review of the backward translations. The purpose of this meeting was to reach a consensus among the translators for a second unified version of the questionnaires in each national language.

##### Pre-testing in target population using probe technique

Prior to proceeding to the validation procedure, the second unified version of the questionnaire was administered to ten parents of patients with JIA and ten children of different educational levels and backgrounds, using a probe technique to ensure parents’ and children’s understanding in the target population. The probe method was carried out as follows: a health professional, familiar with the intent of each question, administered the questionnaires to the parents and patients, asking to consider each question and elucidate their understanding of each item in an open-ended manner. The health professional assessed if the question was perfectly understood by each parent and child. Questions that were misunderstood by more than 20% of the parents or patients were reviewed by the National Coordinating Centre and revised appropriately.

##### Third unified forward version

A third meeting was convened among all the forward and backward translators to take into account the results of the probe technique. The goal of this meeting was to reach a consensus for a final unified version of the questionnaires in each language considered.

In case the JAMAR translation was already available in the local language because the first phase of the project was already completed in a country with the same official language, the PRINTO national coordinator was allowed to test only the available version with the probe technique.

#### Phase II: validation

Following the process of cross-cultural adaptation, a large-scale data collection phase was set up using the third unified forward version of the questionnaires.

Each participating centre was asked to collect demographic, clinical data and the JAMAR in 100 consecutive JIA patients or all consecutive patients seen in a 6-month period and to administer the JAMAR to at least 100 healthy children and their parents.

The validation procedures followed the classical steps of the psychometric theory [19].

##### Demographic and clinical characteristics of the subjects

Descriptive statistics were performed for demographic and clinical variables. Categorical variables were summarized in terms of absolute frequencies and percentages; quantitative variables were reported in terms of medians and first and third quartiles (1st–3rd q). Comparison of quantitative variables between JIA patients and the groups of healthy children was made by the Mann–Whitney *U* test due to the ordinal nature of the data. Comparison of quantitative scores among the JIA subtypes was made by the Kruskal–Wallis test. Correlations were evaluated by the Pearson’s correlation coefficient or by the Spearman’s correlation coefficient. The correlation coefficients were classified as follows: *r* < 0.4 = weak, 0.4–0.59 = moderate, 0.6–0.79 = strong and ≥ 0.80 = very strong correlation [20]. All statistical tests were two-sided and a *P* value < 0.05 was considered as statistically significant.

##### Descriptive analysis of the items

The median and first and third quartiles of missing values and items marked as not applicable for each item have been calculated. The pattern of responses has been also evaluated to determine whether the data will be normally or skewed distributed (e.g. do people report at either extremes of the response continuum?). The means and standard deviations of items within a scale should be roughly equivalent (first Likert assumption); if this assumption is met, then it is possible to avoid a weighting of the items.

Floor and ceiling effects have been performed to check whether scale scores have substantial variability in the population of interest. The floor effect refers to the frequency of the lowest possible responses within an item, while the ceiling effect refers to the frequency of the highest possible responses within an item.

##### Equal item-scale correlation (second Likert assumption)

These should be roughly equivalent for items within a scale when corrected for overlap. This analysis will be carried out using the Pearson correlation coefficients to test the second Likert assumption (equal item–scale correlations); that is, each item should contribute roughly an equal proportion of information to the total score regarding the construct being measured. If the items have roughly equal variances, they do not need to be standardized.

##### Item internal consistency (item–scale correlation corrected for overlap)

It tests the third Likert assumption, e.g. items should be substantially linear related to the total score computed from the items in that scale. It requires a Pearson item correlation of at least 0.4 after correction for item–scale overlap. Items not meeting these criteria might have to be revised. It is considered satisfactory if 90% or more of the items meet these criteria.

##### Internal consistency (Cronbach’s alpha)

It refers to the extent to which the measured variance in a score reflects the true score rather than random error; that is, the extent to which measures give consistent or accurate results. Reliability was measured by Cronbach’s alpha coefficients that is considered acceptable if ≥ 0.7 [21]. Therefore, the items should all measure the same latent construct, and this coefficient gives a measure of the internal consistency; these coefficients were calculated either for subscales or for the entire questionnaire.

##### Interscale correlation

It was tested using the means of Pearson correlation coefficients. It requires that the correlation between two scales is less than their reliability coefficients as measured by Cronbach’s alpha. It can be viewed as a correlation between a scale and itself, and it is used to evaluate how each scale is distinct from other scales [22].

##### Test–retest reliability

It is a test of stability and represents the reproducibility within the same individual 1–2 weeks apart from the first administration of the questionnaire. The intra-class correlation coefficient (ICC) was classified as follows: ICC < 0.2 = poor, 0.2–0.39 = fair, 0.4–0.59 = moderate, 0.6–0.79 = substantial and ≥ 0.80 = almost perfect reproducibility [21].

##### Convergent validity

It is the correlation of the summary scores with external criterion variables not used to score the scales. This was done by assessing the Spearman correlation coefficients of the PF total score, the PhS and PsS subscales of the HRQoL section and the 21-circle VAS for pain, disease activity, and well-being with the other variables included in the core set variables for JIA, that are the physician’s evaluation of current disease activity, the number of joints with active arthritis, the number of joints with limited range of motion, and the ESR [23]. Correlations of PF and HRQoL scores with the VAS included in the JAMAR were also assessed.

*Software* All data were analysed using SAS 9.3 (Institute Inc., Cary, NC, USA) software.

## Results

A total of 13,843 subjects were enrolled from 49 countries: Algeria, Argentina, Belgium, Brazil, Bulgaria, Canada, Chile, Colombia, Croatia, Czech Republic, Denmark, Ecuador, Egypt, Estonia, Finland, France, Georgia, Germany, Greece, Hungary, India, Islamic Republic of Iran, Israel, Italy, Latvia, Libya, Lithuania, Mexico, Netherlands, Norway, Oman, Paraguay, Poland, Portugal, Romania, Russian Federation, Saudi Arabia, Serbia, Slovakia, Slovenia, South Africa, Spain, Sweden, Switzerland, Thailand, Turkey, Ukraine, United Kingdom and United States of America. As shown in Table 1 9021 patients had JIA (10.7% with systemic arthritis, 41.9% with oligoarthritis, 23.5% with RF negative polyarthritis, 4.2% with RF positive polyarthritis, 3.4% with psoriatic arthritis, 10.6% with enthesitis-related arthritis and 5.7% with undifferentiated arthritis) while 4822 were healthy children.

**Table 1:** Number of patients with JIA (frequency in brackets) and number of healthy children collected in the 49 countries participating in Phase I (cross-cultural adaptation) and Phase II (data collection and validation)

Countries	Systemic	Oligoarthritis	RF − polyarthritis	RF + polyarthritis	Psoriatic arthritis	Enthesitis-related arthritis	Undifferentiated arthritis	All JIA patients	Healthy
Algeria	7 (10%)	25 (36%)	15 (21%)	14 (20%)	1 (1%)	8 (12%)	0 (0%)	70	70
Argentina	86 (23%)	115 (31%)	105 (28%)	37 (10%)	5 (1%)	23 (6%)	2 (1%)	373	100
Belgium	8 (8%)	33 (33%)	24 (24%)	2 (2%)	6 (6%)	13 (13%)	14 (14%)	100	99
Brazil	34 (15%)	100 (43%)	52 (22%)	11 (5%)	4 (2%)	25 (11%)	5 (2%)	231	72
Bulgaria	22 (12%)	98 (53%)	43 (23%)	3 (2%)	3 (2%)	9 (5%)	5 (3%)	183	100
Canada	6 (3%)	87 (41%)	58 (28%)	6 (3%)	9 (4%)	22 (11%)	20 (10%)	208	152
Chile	6 (12%)	12 (25%)	11 (22%)	1 (2%)	0 (0%)	4 (8%)	15 (31%)	49	70
Colombia	2 (9%)	3 (14%)	6 (27%)	3 (14%)	0 (0%)	8 (36%)	0 (0%)	22	0
Croatia	7 (7%)	38 (38%)	19 (19%)	5 (5%)	0 (0%)	12 (12%)	19 (19%)	100	100
Czech Republic	6 (6%)	37 (36%)	39 (38%)	1 (1%)	3 (3%)	13 (12%)	4 (4%)	103	100
Denmark	24 (8%)	106 (35%)	67 (22%)	17 (6%)	19 (6%)	39 (13%)	31 (10%)	303	99
Ecuador	4 (17%)	1 (4%)	4 (17%)	4 (17%)	0 (0%)	2 (9%)	8 (36%)	23	23
Egypt	20 (20%)	10 (10%)	24 (24%)	2 (2%)	2 (2%)	2 (2%)	40 (40%)	100	100
Estonia	0 (0%)	79 (72%)	20 (18%)	2 (2%)	2 (2%)	2 (2%)	5 (4%)	110	98
Finland	2 (1%)	80 (46%)	69 (40%)	1 (1%)	1 (1%)	13 (7%)	7 (4%)	173	100
France	23 (23%)	45 (45%)	20 (20%)	3 (3%)	0 (0%)	5 (5%)	4 (4%)	100	122
Georgia	26 (26%)	57 (57%)	16 (16%)	1 (1%)	0 (0%)	0 (0%)	0 (0%)	100	100
Germany	9 (3%)	117 (37%)	75 (23%)	10 (3%)	18 (6%)	65 (20%)	25 (8%)	319	100
Greece	16 (6%)	157 (57%)	58 (21%)	1 (1%)	8 (3%)	16 (6%)	16 (6%)	272	100
Hungary	8 (4%)	85 (41%)	58 (28%)	4 (2%)	10 (5%)	33 (16%)	8 (4%)	206	90
India	78 (28%)	30 (11%)	38 (14%)	19 (7%)	5 (2%)	88 (32%)	17 (6%)	275	98
Iran	15 (15%)	69 (67%)	16 (16%)	1 (1%)	1 (1%)	0 (0%)	0 (0%)	102	198
Israel	20 (17%)	65 (56%)	24 (20%)	1 (1%)	2 (2%)	3 (3%)	1 (1%)	116	98
Italy	93 (7%)	772 (60%)	277 (21%)	18 (1%)	49 (4%)	45 (4%)	42 (3%)	1296	100
Latvia	2 (2%)	56 (56%)	17 (17%)	13 (13%)	4 (4%)	7 (7%)	1 (1%)	100	204
Libya	22 (22%)	26 (26%)	25 (25%)	5 (5%)	4 (4%)	13 (13%)	5 (5%)	100	100
Lithuania	6 (6%)	39 (38%)	24 (24%)	1 (1%)	15 (15%)	15 (15%)	1 (1%)	101	116
Mexico	16 (16%)	16 (16%)	20 (20%)	30 (30%)	1 (1%)	16 (16%)	1 (1%)	100	99
Netherlands	30 (14%)	83 (40%)	54 (26%)	9 (4%)	9 (4%)	12 (6%)	12 (6%)	209	107
Norway	10 (3%)	124 (41%)	78 (26%)	10 (3%)	12 (4%)	27 (10%)	40 (13%)	301	74
Oman	13 (23%)	16 (28%)	20 (35%)	6 (10%)	2 (4%)	0 (0%)	0 (0%)	57	85
Paraguay	1 (2%)	14 (27%)	19 (37%)	9 (18%)	3 (6%)	5 (10%)	0 (0%)	51	100
Poland	16 (10%)	77 (50%)	38 (25%)	9 (6%)	0 (0%)	8 (5%)	6 (4%)	154	91
Portugal	5 (6%)	55 (69%)	3 (4%)	3 (4%)	0 (0%)	11 (13%)	3 (4%)	80	30
Romania	37 (12%)	67 (22%)	99 (32%)	29 (9%)	6 (2%)	62 (20%)	10 (3%)	310	100
Russian Federation	25 (25%)	19 (19%)	38 (38%)	3 (3%)	0 (0%)	15 (15%)	0 (0%)	100	198
Saudi Arabia	27 (27%)	23 (23%)	25 (25%)	13 (13%)	6 (6%)	3 (3%)	3 (3%)	100	100
Serbia	13 (5%)	110 (44%)	59 (24%)	11 (4%)	6 (3%)	39 (16%)	10 (4%)	248	100
Slovakia	6 (6%)	42 (39%)	33 (30%)	4 (4%)	0 (0%)	14 (13%)	9 (8%)	108	100
Slovenia	7 (7%)	47 (47%)	22 (22%)	2 (2%)	4 (4%)	9 (9%)	9 (9%)	100	120
South Africa	4 (4%)	32 (35%)	21 (23%)	6 (7%)	6 (7%)	14 (15%)	8 (9%)	91	98
Spain	45 (9%)	260 (49%)	96 (18%)	5 (1%)	29 (5%)	50 (10%)	41 (8%)	526	78
Sweden	6 (9%)	30 (44%)	9 (13%)	2 (3%)	3 (4%)	8 (12%)	10 (15%)	68	76
Switzerland	3 (3%)	43 (44%)	16 (16%)	0 (0%)	2 (2%)	25 (26%)	9 (9%)	98	64
Thailand	47 (45%)	11 (11%)	10 (9%)	11 (11%)	0 (0%)	25 (24%)	0 (0%)	104	102
Turkey	64 (14%)	189 (41%)	105 (22%)	11 (2%)	15 (3%)	70 (15%)	12 (3%)	466	93
Ukraine	12 (12%)	44 (44%)	20 (20%)	3 (3%)	1 (1%)	16 (16%)	4 (4%)	100	100
United Kingdom	7 (7%)	38 (38%)	27 (27%)	1 (1%)	5 (5%)	9 (9%)	13 (13%)	100	100
USA	16 (5%)	98 (31%)	107 (34%)	16 (5%)	28 (9%)	34 (11%)	16 (5%)	315	98
Total (*N* = 49 countries)*	962	3780	2123	379	309	957	511	9021	4822

*Additional 3 countries (Albania, Costa Rica and Peru) participated only in Phase I (cross-cultural adapation)

In Table 2 is reported the list of PRINTO National Coordinators who oversaw the cross-cultural adaptation phase and the data collection for the validation phase in the respective countries, while in Table 3 is reported the list of PRINTO Directors who oversaw the data collection for the validation phase in their respective centres.

**Table 2:** List of the 54 country-specific principle investigators (51 PRINTO National Coordinators and 3 representatives for Canada and USA) who oversaw the Phase I (cross-cultural adaptation) and the Phase II (data collection and validation) in their respective countries

First name	Last name	Institution	Town	Country
Anuela	Kondi	University Hospital Centre	Tirana	Albania
Maya-Feriel	Aiche	Établissement Hospitalier Spécialisé (EHS Douera), Department of Rheumatology	Alger	Algeria
Stella Maris	Garay	Hospital Sor María Ludovica	La Plata	Argentina
Dehoorne	Joke	Gent University Hospital	Gent	Belgium
Claudia	Saad Magalhaes	Hospital das Clínicas - Botucatu Medicine University, UNESP	Botucatu	Brazil
Dimitrina	Mihaylova	University Children Hospital	Sofia	Bulgaria
Paivi	Miettunen	Alberta Children’s Hospital	Calgary	Canada
Gaelle	Chédeville	The Montreal Children’s Hospital	Montreal	Canada
Ximena	Norambuena	Hospital Dr. Exequiel Gonzalez Cortes	Santiago	Chile
Clara	Malagon	Hospital Universitario Simon Bolivar	Bogota	Colombia
Olga	Arguedas	Hospital Nacional De Ninos Dr. Carlos Saenz Herrera	San Jose	Costa Rica
Miroslav	Harjacek	Clinical Hospital Center Sestre Milosrdnice	Zagreb	Croatia
Pavla	Dolezalova	Charles University in Prague and General University Hospital	Praha	Czech Republic
Susan	Nielsen	Rigshospitalet	Copenhagen	Denmark
Cristina	Herrera Mora	Hospital de Niños Roberto Gilbert Elizalde	Guayaquil	Ecuador
Yasser	El Miedany	Ain Shams University	Cairo	Egypt
Chris	Pruunsild	Tartu University Hospital, Children’s Clinic	Tartu	Estonia
Pekka	Lahdenne	Children’s Hospital, Helsinki University Central Hospital	Helsinki	Finland
Pierre	Quartier	Paris-Descartes University, IMAGINE Institute, Necker Children’s Hospital	Paris	France
Maka	Ioseliani	M. Iashvili Children’s Central Clinic	Tbilisi	Georgia
Dirk	Foell	University Hospital Muenster	Muenster	Germany
Maria	Trachana	Hippokration General Hospital, Thessaloniki University School of Medicine	Thessaloniki	Greece
Ilonka	Orban	National Institute of Rheumatology and Physiotherapy	Budapest	Hungary
Amita	Aggarwal	Sanjay Gandhi Postgraduate Institute of Medical Sciences	Lucknow	India
Nahid	Shafaie	Shariati Hospital	Teheran	Islamic Republic of Iran
Yosef	Uziel	Meir Medical Centre	Kfar Saba	Israel
Alessandro	Consolaro	IRCCS Giannina Gaslini	Genoa	Italy
Ingrida	Rumba-Rozenfelde	University of Latvia	Riga	Latvia
Soad	Hashad	Tripoli Children’s Hospital	Tripoli	Libya
Violeta	Panaviene	Vilnius University	Vilnius	Lithuania
Ruben	Burgos-Vargas	Hospital General de Mexico	Mexico City	Mexico
Nico	Wulffraat	Wilhelmina Kinderziekenhuis	Utrecht	Netherlands
Berit	Flato	Oslo University Hospital	Oslo	Norway
Safiya	Al-Abrawi	Royal Hospital	Muscat	Oman
Zoilo	Morel Ayala	Centro Materno Infantil. Hospital De Clinicas. Universidad Nacional De Asuncion	San Lorenzo	Paraguay
Amparo	Ibanez Estrella	National Institute Salud del Nino	Breña, Lima	Peru
Lidia	Rutkowska-Sak	Institute of Rheumatology	Warsaw	Poland
Jose Antonio	Melo-Gomes	Portuguese Institute of Rheumatology	Lisbon	Portugal
Calin	Lazar	Children Emergencies Hospital	Cluj-Napoca	Romania
Irina	Nikishina	V.A. Nasonova Research Institute of Rheumatology	Moscow	Russian Federation
Sulaiman M	Al-Mayouf	King Faisal Specialist Hospital and Research Center	Riyadh	Saudi Arabia
Gordana	Susic	Institute of Rheumatology, Belgrade	Belgrade	Serbia
Veronika	Vargova	Faculty of Medicine, Pavol Jozef Šafárik University in Košice	Kosice	Slovakia
Tadej	Avcin	University Children’s Hospital, University Medical Centre Ljubljana	Ljubljana	Slovenia
Christiaan	Scott	Red Cross Children Hospital and Groote Schuur Hospital	Cape Town	South Africa
Jaime	De Inocencio	Hospital Universitario 12 de Octubre, and Universidad Complutense de Madrid	Madrid	Spain
Boel	Andersson Gare	Ryhov County Hospital	Jonkoping	Sweden
Michael	Hofer	Centre Multisite Romand de Rhumatologie Pediatrique/Centre Hospitalier Universitaire Vaudois (CHUV)	Lausanne	Switzerland
Traudel	Saurenmann	Kantonsspital Winterthur	Winterthur	Switzerland
Soamarat	Vilaiyuk	Ramathibodi Hospital	Bangkok	Thailand
Erkan	Demirkaya	Antalya Life Hospital	Antalya	Turkey
Yaryna	Boyko	Western Ukrainian Specialized Children’s Hospital	Lviv	Ukraine
Neil	Martin	The Royal Hospital for Children	Glasgow	United Kingdom
Dan	Lovell	Cincinnati Children’s Hospital Medical Center	Cincinnati, OH	United States

**Table 3:** List of 60 additional local investigators (58 PRINTO Directors and 2 representatives for USA) who oversaw the Phase II of the project (data collection and validation) at their respective centres

First name	Last name	Institution	Town	Country
Ruben	Cuttica	Hospital Pedro de Elizalde	Buenos Aires	Argentina
Carmen	De Cunto	Buenos Aires Italy Hospital	Buenos Aires	Argentina
Graciela	Espada	Hospital de Ninos Ricardo Gutierrez	Buenos Aires	Argentina
Maria Martha	Katsicas	Hospital de Pediatria Juan P. Garrahan	Buenos Aires	Argentina
Carine	Wouters	University Hospital Gasthuisberg	Leuven	Belgium
Sheila	K. Oliveira	Universidade Federal do Rio de Janeiro	Rio de Janeiro	Brazil
Flavio	Sztajnbok	University Hospital Pedro Ernesto	Rio de Janeiro	Brazil
Boriana	Varbanova	Varna Medical University	Varna	Bulgaria
Troels	Herlin	Skejby Sygehus, Aarhus University Hospital	Aarhus	Denmark
Anne	Estmann	Odense University Hospital	Odense	Denmark
Liisa	Kroger	Kuopio University Hospital	Kuopio	Finland
Paula	Vahasalo	Oulu University Hospital	Oulu	Finland
Merja	Malin	Tampere University Hospital	Tampere	Finland
Anne	Putto-Laurila	Turku University Hospital	Turku	Finland
Karaman	Pagava	Tbilisi State Medical University Pediatric Clinic	Tbilisi	Georgia
Nikolay	Tzaribachev	Pediatric Rheumatology Research Institute GmbH	Bad Bramstedt	Germany
Kirsten	Minden	Charite University Hospital Berlin	Berlin	Germany
Hans-Iko	Huppertz	Clinic Bremen-Mitte, Prof.-Hesse Children’s Hospital	Bremen	Germany
Johannes-Peter	Haas	German Center for Pediatric and Adolescent Rheumatology	Garmisch-Partenkirchen	Germany
Ivan	Foeldvari	Hamburg Centre for Pediatric and Adolescent Rheumatology	Hamburg	Germany
Gerd	Horneff	Asklepios Children’s Hospital Sankt Augustin	Sankt Augustin	Germany
Gerd	Ganser	St. Josef-Stift Sendenhorst	Sendenhorst	Germany
Elena	Tsitsami	University of Athens Medical School, Children Hospital Aghia Sophia	Athens	Greece
Olga	Vougiouka	“P. & A. Kyriakou” Children’s Hospital	Athens	Greece
Tamas	Constantin	Semmelweis University	Budapest	Hungary
Raju	Khubchandani	Jaslok Hospital and Research Centre	Mumbai	India
Sujata	Sawhney	Sir Ganga Ram Hospital Marg	New Delhi	India
Yahya	Aghighi	Vali-e-Asr Children’s Hospital	Teheran	Islamic Republic of Iran
Mohammad Hasan	Moradinejad	Children’s Hospital, Medical Center	Teheran	Islamic Republic of Iran
Liora	Harel	Schneider Childrens Medical Center	Petach-Tikvah	Israel
Angela	Miniaci	Azienda Ospedaliero-Universitaria S.Orsola-Malpighi	Bologna	Italy
Francesco	La Torre	Ospedale Antonio Perrino	Brindisi	Italy
Rosa Anna	Podda	Ospedale Regionale Microcitemia—II Clinica Pediatrica	Cagliari	Italy
Patrizia	Barone	Catania University Hospital	Catania	Italy
Elisabetta	Cortis	Santa Maria della Stella Hospital	Ciconia	Italy
Rolando	Cimaz	University Hospital Meyer	Firenze	Italy
Romina	Gallizzi	Università di Messina	Messina	Italy
Fabrizia	Corona	Fondazione IRCCS Ca’ Granda-Ospedale Maggiore Policlinico	Milano	Italy
Valeria	Gerloni	Istituto Gaetano Pini	Milano	Italy
Maria Cristina	Maggio	Children Hospital	Palermo	Italy
Rita	Consolini	Santa Chiara Hospital, University of Pisa	Pisa	Italy
Fabrizio	De Benedetti	Bambino Gesù Children’s Hospital	Roma	Italy
Donato	Rigante	Cattolica Sacro Cuore University	Roma	Italy
Silvana	Martino	Paediatrics, University of Torino	Torino	Italy
Adele	Civino	Cardinale G. Panico Hospital	Tricase	Italy
Serena	Pastore	IRCCS Burlo Garofolo	Trieste	Italy
Sara	Pieropan	G.B. Rossi	Verona	Italy
Sylvia	Kamphuis	ErasmusMC Sophia Childrens Hospital	Rotterdam	Netherlands
Ellen Berit	Nordal	University Hospital of Northern Norway	Tromso	Norway
Marite	Rygg	St. Olavs University Hospital of Trondheim	Trondheim	Norway
Reem	Abdwani	Sultan Qaboos University Hospital	Muscat	Oman
Elzbieta	Smolewska	Medical University of Łódź	Lodz	Poland
Adriana	Apostol	County Emergency Hospital	Constanta	Romania
Constantin	Ailioaie	Children Emergencies Hospital	Iasi	Romania
Matilda	Laday	County Emergency Hospital	Tirgu-Mures	Romania
Ekaterina	Alexeeva	Federal State Autonomous Institution “National Medical Research Center of Children’s Health” of the Ministry of Health of the Russian Federation	Moscow	Russian Federation
Jelena	Vojinovic	University of Nis, Faculty of Medicine, and Clinical Center Nis	Nis	Serbia
Gordana	Vijatov-Djuric	Institute for Child and Youth Health Care of Vojvodina	Novi Sad	Serbia
Tomas	Dallos	Comenius University Medical School	Bratislava	Slovakia
Jordi	Anton Lopez	Hospital Sant Joan de Déu, University of Barcelona	Barcelona	Spain
Alina Lucica	Boteanu	University Hospital Ramón y Cajal	Madrid	Spain
Rosa	Merino	Hospital Universitario La Paz	Madrid	Spain
Pablo	Mesa-del-Castillo	Hospital Clínico Universitario Virgen de la Arrixaca	Murcia	Spain
Inmaculada	Calvo Penades	University Hospital La Fe	Valencia	Spain
Lillemor	Berntson	Uppsala University Hospital	Uppsala	Sweden
Erbil	Unsal	Dokuz EyluL University Medical Faculty	Balcova, Izmir	Turkey
Nuray	Aktay Ayaz	Kanuni Sultan Süleyman Education and Research Hospital	Istanbul	Turkey
Ozgur	Kasapcopur	Istanbul University, Cerrahpasa Medical Faculty	Istanbul	Turkey
Pamela	Weiss	Children’s Hospital of Philadelphia	Philadelphia, PA	United States
Sarah	Ringold	Seattle Children’s Hospital	Seattle, WA	United States

### JAMAR cross-cultural adaptation

The current supplement contains the cross-cultural adaptation and validation of the JAMAR for the 49 countries listed above (see Table 1).

Two forward and two backward translations were carried out for 22 countries: Argentina, Chile, Croatia, Czech Republic, Denmark, Egypt, Estonia, Georgia, Germany, Iran, Israel, Latvia, Lithuania, Netherlands, Norway, Romania, Slovakia, South Africa, Sweden, Switzerland, Thailand and Ukraine. For Greece, two forward translations and three backward translations were available. Three forward and two backward translations were obtained for 14 countries: Brazil, Bulgaria, Finland, France, India, Italy, Mexico, Poland, Portugal, Russian Federation, Saudi Arabia, Serbia, Slovenia and Turkey. For Hungary and Spain, three forward and three backward translations were done.

For some countries with similar languages there was only an adaptation of the languages with the changing of words whose use is different. This was done for ten countries: Algeria, Libya and Oman from the Saudi Arabia version, Paraguay from Mexican Spanish version, Flemish from Dutch, Ecuadorian Spanish from Argentinian Spanish, Colombian Spanish from Castilian Spanish, Swiss German from German, Canadian French from Swiss French, American and Canadian English from the British English version.

For Albania, Costa Rica (adapting from Castilian Spanish version), German-speaking Switzerland (adapting from German) and Peru (adapting from Mexican Spanish version), the translation phase was completed but the data collection of JIA patients and healthy children was not performed.

### JAMAR validation

The results of the JAMAR validation pertaining to each country are fully described in each of the accompanying manuscripts of the supplement.

According to the results of the validation analysis implemented for each participating country, the parent and patient versions of the JAMAR possess satisfactory psychometric properties. The disease-specific components of the questionnaire discriminated well between patients with JIA and healthy controls. Generally, the results obtained for the parent version of the JAMAR are similar to those obtained for the child version, which suggests that children are equally reliable proxy reporters of their disease and health status as their parents. The JAMAR was found to have satisfactory psychometric properties and it is thus a reliable and effective tool for the multidimensional assessment of children with JIA.

### Final remarks

The results of the present study show that the cross-cultural adaptation is a valid process to obtain reliable instruments to be used in the different socio-economic realities of the countries participating in the project.

The process of cross-cultural adaptation refers to the measurement of the same phenomenon in different cultures using the same instruments and should be distinguished from the concept of cross-cultural comparison that refers to comparative studies of a phenomenon across cultures aimed at identifying differences attributable to cultures. It was decided to follow the guidelines proposed by Guillemin et al. [18] for the cross-cultural adaptation procedures to have a standardized approach that was easily applicable to all the countries members of PRINTO.

For the countries sharing similar languages, the questionnaires were adapted from the original mother tongue country (i.e. the version for Paraguay, Oman and USA were derived from the Mexican Spanish, the Saudi Arabic and the British English versions, respectively); this process required merely a change of certain words whose use is different in these linguistic varieties. In all the other countries, the guidelines proved both easy to apply and reliable, with the backward translations revealing that the concepts most difficult to render in the target language were the categories of answers to the items and the instructions statement. For all the other concepts there was an excellent concordance between the backward translations and the original standard English version, indicating the reliability of the method.

All translations presented in this supplement have been evaluated using traditional multi-trait item scaling analysis.

In conclusion, PRINTO has cross-culturally adapted and evaluated the JAMAR in 54 languages across 52 different countries. The JAMAR proved to be a reliable multidimensional tool for the assessment of patient with JIA. Use of well-evaluated translations allows for the standardized assessment of children with JIA.

The manuscripts in this supplement present the preliminary psychometric findings of the cross-cultural adaptation and psychometric evaluation of the JAMAR for the 49 countries that took part both in the cross-cultural translation phase and in the related data collection.
